# Gaston’s Operation

**Published:** 1887-12

**Authors:** 


### GASTON'S OPERATION.

In the *Medical Record*, of New York, for the 12th instant, it is observed that Professor Kappeler has operated successfully upon the human subject for the union of the gall bladder and duodenum, thus placing Gaston's operation on a practicable basis before the profession.

It is likely that the more simple process of uniting the walls by a single loop of suture will come to be adopted instead of that by incision and suture as resorted to by Kappeler.

We have to note that Professor Gaston performed ovariotomy with success on the 5th instant, upon a lady in this city, being assisted by Drs. Bizzell, Roy, Parks, and Earnest. A large multilocular cystic tumor of one ovary and cystic degeneration of the other required the removal of both, the pedicles being ligated with iron-died silk, divided by the thermo cautery and dropped. The opening in the peritoneum was closed by continuous catgut suture, while that in the abdominal walls was secured by eleven stitches of iron-dyed silk. The line of incision in the linea alba was dusted with iodoform and the abdomen was covered with a compress of antiseptic gauze and cotton, having a broad bandage placed firmly around the body. There was no suppuration from the suture, six stiches being removed on the seventh and the remaining five on the tenth day, with union by first intention of the entire incision, reaching from the umbilicus to the mons veneris.

The patient is now on the twentieth day, sitting up a few hours each day, pulse and temperature normal, while she eats and sleeps as if in perfect health, with a good prospect of complete recovery.

The operation was done throughout with antiseptic precautions.

